# Extracorporeal shockwave therapy versus sham extracorporeal shockwave therapy for chronic Achilles tendinopathy: a meta-analysis of randomized controlled trials

**DOI:** 10.7717/peerj.20506

**Published:** 2026-01-06

**Authors:** Tingting Ni, Yanmin Zhao, Long Pang, Weili Fu

**Affiliations:** 1 Operating Room of Anesthesia Surgery Center, West China Hospital, Sichuan University, Chengdu, Sichuan, China; 2 West China School of Nursing, Sichuan University, Chengdu, Sichuan, China; 3 Outpatient Department, West China Hospital, Sichuan University, Chengdu, Sichuan, China; 4 Sports Medicine Center, West China Hospital, Sichuan University, Chengdu, Sichuan, China; 5 Department of Orthopedics, Orthopedic Research Institute, West China Hospital, Sichuan University, Chengdu, Sichuan, China

**Keywords:** Achilles tendinopathy, Extracorporeal shockwave therapy, Placebos, Meta-analysis, Randomized controlled trial

## Abstract

**Background:**

Chronic Achilles tendinopathy is a persistent and debilitating condition. Extracorporeal shockwave therapy (ESWT) is widely used, but its true effectiveness and safety for chronic Achilles tendinopathy remains debated. This study aimed to compare the effectiveness and safety of ESWT and sham ESWT for chronic Achilles tendinopathy.

**Methods:**

Following Preferred Reporting Items for Systematic Reviews and Meta-Analyses (PRISMA) guidelines, PubMed, EMBASE, Cochrane Library, and Web of Science were systematically searched for randomized controlled trials (RCTs) comparing ESWT with sham ESWT in chronic Achilles tendinopathy. Primary outcomes included pain reduction (change in Visual Analog Scale (ΔVAS), minimal clinically important difference (MCID) 1.1/10) and functional improvement (change in Victorian Institute of Sports Assessment-Achilles (ΔVISA-A), MCID 8/100; change in American Orthopaedic Foot and Ankle Society (ΔAOFAS), MCID 12/100). Secondary outcomes encompassed adverse events. Risk of bias was assessed via Risk of Bias 2 (RoB2). Meta-analysis used RevMan 5.4.1, with weighted mean differences (WMD) and odds ratios (ORs) for continuous and dichotomous outcomes, respectively. Hypothesis-generating subgroup analyses explored ESWT type, tendinopathy classification, and symptom duration.

**Results:**

Eight RCTs (458 participants) were included. No significant differences were observed between ESWT and sham groups in ΔVAS or ΔVISA-A, ΔAOFAS across follow-ups all *P* > 0.05). Adverse events were higher with ESWT (4.5% *vs.* 1.2%), though non-significant (*P* = 0.12). Subgroup analysis found that ESWT led to significant VAS reduction in patients with symptom duration < 12 months at 1-month (WMD: −0.76, *P* = 0.03) and 3-month (WMD: −1.23, *P* = 0.001) follow-ups than Sham ESWT.

**Conclusions:**

ESWT showed no clear overall benefits compared to sham ESWT for chronic Achilles tendinopathy, but exploratory analyses hint at possible short-term pain relief for patients with symptoms lasting less than 12 months. More high-quality evidence is needed.

## Introduction

Chronic Achilles tendinopathy, defined as symptoms persisting over three months, is characterized by localized pain, morning stiffness, and functional impairment (Opdam et al., 2021; Van Dijk et al., 2011). It presents as either insertional (at the calcaneal attachment) or non-insertional (2–6 cm proximal to insertion), with distinct clinical characteristics (Chen et al., 2022). The condition affects 6% of the general population over a lifetime, but its prevalence rises significantly to 52% among endurance runners (Chen et al., 2024; Kujala, Sarna & Kaprio, 2005). It predominantly impacts middle-aged individuals engaged in sports, though sedentary populations with risk factors such as obesity and diabetes remain susceptible (Van der Vlist et al., 2019). Chronic Achilles tendinopathy significantly impacts quality of life, work productivity, and healthcare costs, with annual expenses averaging €840 per patient (Sleeswijk Visser et al., 2021). Additionally, 25%–60% of patients experience symptoms for 5–10 years (De Vos et al., 2021; Silbernagel, Hanlon & Sprague, 2020), making it a persistent and disabling condition that frequently drives clinical visits.

Conservative treatments for chronic Achilles tendinopathy mainly include activity modification, eccentric loading exercises, orthotic devices, non-steroidal anti-inflammatory drugs, and various physical therapy modalities (Chen et al., 2022; De Vos et al., 2021; Silbernagel, Hanlon & Sprague, 2020; Tarantino et al., 2023). However, these conventional treatments fail to achieve satisfactory results in about 25–45% of patients, often requiring additional interventions (De Vos et al., 2021; Silbernagel, Hanlon & Sprague, 2020). Furthermore, placebo effects have been widely documented in the conservative management of musculoskeletal conditions (Acosta-Olivo et al., 2020; Hafliðadóttir et al., 2021; Previtali et al., 2021), with a recent review (Viglione et al., 2023) reporting statistically and clinically significant placebo responses in the treatment of plantar fasciitis, especially in pain relief. This highlights a critical challenge in evaluating treatment effectiveness, particular for pain-related outcomes.

Extracorporeal Shockwave Therapy (ESWT) has emerged as a promising non-invasive treatment option for tendinopathy, involving the application of high-energy acoustic waves to affected tissues (Majidi et al., 2024). ESWT is theorized to stimulate tissue healing through multiple mechanisms, including neovascularization, enhanced blood supply, increased growth factor expression, anti-inflammatory effects, and stimulation of collagen synthesis (Poenaru, Sandulescu & Cinteza, 2023; Ryskalin et al., 2022; Simplicio et al., 2020). Two primary types of ESWT are utilized clinically: focused ESWT (fESWT), which delivers concentrated energy to deeper tissues, and radial ESWT (rESWT), which delivers more dispersed energy affecting larger, superficial areas (Auersperg & Trieb, 2020). The non-invasive nature of ESWT, coupled with its relatively low risk profile and potential to avoid surgery, has garnered significant clinical interest.

Despite its widespread adoption, evidence of ESWT’s effectiveness for chronic Achilles tendinopathy remains controversial. Previous systematic reviews and meta-analyses have reported inconsistent findings (Charles et al., 2023; Fan et al., 2020; Feeney, 2022; Paantjens et al., 2022; Stania et al., 2019). These discrepancies may stem from methodological heterogeneity, including variations in control groups (ranging from wait-and-see approaches to active therapies), treatment protocols, and outcome assessments. Additionally, concerns regarding potential adverse effects such as pain during application, local edema, erythema, and rare cases of tendon rupture warrant careful consideration (Majidi et al., 2024; Simplicio et al., 2020). Notably, previous reviews have compared ESWT to heterogeneous control interventions rather than specifically to sham treatments, making it challenging to distinguish true therapeutic effects from placebo responses. This represents a significant gap in the literature, as placebo effects are particularly relevant in pain-related conditions and subjective outcome measures.

This meta-analysis aims to assess the effectiveness and safety of ESWT compared exclusively to sham ESWT for chronic Achilles tendinopathy. By focusing on randomized controlled trials (RCTs) with sham controls, we seek to isolate ESWT’s specific therapeutic effect beyond placebo effects. Based on established minimal clinically important difference (MCID) thresholds (Challoumas et al., 2023; Murphy et al., 2018), we hypothesize that ESWT may demonstrate superior pain reduction, assessed by the Visual Analog Scale (VAS) with an MCID of 1.1 out of 10, and functional improvement, evaluated using the Victorian Institute of Sports Assessment-Achilles (VISA-A) score with an MCID of eight out of 100 and the American Orthopaedic Foot and Ankle Society (AOFAS) score with an MCID of 12 out of 100, while maintaining an acceptable safety profile compared to sham ESWT.

## Materials & Methods

This meta-analysis adhered to the Preferred Reporting Items for Systematic Reviews and Meta-Analyses (PRISMA) guidelines (Liberati et al., 2009) and was preregistered in the International Prospective Register of Systematic Reviews (PROSPERO) with the registration ID: CRD42025648082.

### Literature search

A comprehensive search was conducted across PubMed, EMBASE, the Cochrane Library, and Web of Science databases by two independent reviewers (T.N. and Y.Z.), including all records available up to February 20, 2025. The search strategy used terms including (“Extracorporeal Shockwave Therapy” OR “ESWT” OR “shockwave therapy”) AND (“sham ESWT” OR “placebo ESWT” OR “sham therapy”) AND (“Achilles tendinopathy” OR “chronic Achilles tendinopathy” OR “Achilles tendonitis” OR “tendinosis”). Search queries were adapted to the specific requirements of each database. Discrepancies between the two independent reviewers were resolved through consultation with a third reviewer (W.F.), an orthopedic physician with extensive experience in evidence-based medicine research.

### Inclusion and exclusion criteria

Inclusion Criteria: (1) studies involving adult patients diagnosed with chronic Achilles tendinopathy (duration ≥3 months); (2) studies directly comparing ESWT with sham ESWT; (3) RCTs.

Exclusion Criteria: (1) studies involving acute Achilles tendinopathy, tendon rupture, partial tears, or systemic inflammatory diseases; (2) studies combining ESWT with other active interventions (such as injections) or lacking a sham control; (3) non-RCT designs or studies without detailed ESWT/sham protocol descriptions.

### Data extraction

Data extraction was independently performed by two reviewers (T.N. and Y.Z.), with discrepancies resolved through discussion with a third reviewer (W.F.). Extracted study characteristics included the first author’s name, year of publication, study location, sample size, patient demographics (age, gender, and duration of symptoms), ESWT protocol details (type of ESWT, frequency, intensity, total shocks, sessions, interval, and duration), sham ESWT protocol specifics, blinding and co-intervention details.

The primary outcomes were pain reduction, assessed using the change (endpoint values –baseline values) in VAS (ΔVAS), and functional improvement, evaluated with the change in VISA-A score (ΔVISA-A) (Robinson et al., 2001) and AOFAS score (ΔAOFAS) (Cöster et al., 2014). Secondary outcomes included adverse events related to ESWT or sham ESWT treatments. Based on previous literature (Challoumas et al., 2023; Murphy et al., 2018), the MCID thresholds were set as a reduction of 1.1 points on the VAS (0–10), improvements of eight points on the VISA-A (0–100), and 12 points on the AOFAS score (0–100). The primary outcomes and secondary outcomes constituted the primary findings, while further subgroup analyses (pre-specified for symptom duration, ESWT type, and tendinopathy classification) were treated as hypothesis-generating findings due to their exploratory nature.

### Quality assessment

Two investigators independently evaluated the methodological quality of included studies using the revised Cochrane Risk of Bias Tool (RoB 2) for RCTs (Sterne et al., 2019). This tool assesses bias across five domains: randomization process, deviations from intended interventions, missing outcome data, outcome measurement, and selection of reported results. Each domain was rated as low risk, high risk, or some concerns, following RoB 2 guidelines. The quality of evidence for primary findings was rated using the Grading of Recommendations, Assessment, Development, and Evaluations (GRADE) approach, starting from high quality for RCTs and potentially downgraded based on risk of bias, inconsistency, indirectness, imprecision, and publication bias, as per the GRADE handbook. Publication bias was assessed *via* funnel plots and Egger’s test for outcomes with ten or more studies.

### Statistical analysis

We performed meta-analyses employing Review Manager (RevMan) software, version 5.4.1 (developed by The Cochrane Collaboration in Oxford, UK). For continuous variables, we calculated weighted mean differences (WMDs) accompanied by 95% confidence intervals (CIs), whereas dichotomous variables were summarized using pooled odds ratios (ORs) along with their 95% CIs. The heterogeneity was assessed through the Cochrane Q test and the I^2^ value. A fixed-effects approach was adopted when I^2^ fell below 50%, while a random-effects approach was utilized when I^2^ reached or exceeded 50%. To assess the robustness of the primary findings, sensitivity analyses were performed by excluding trials with significantly different ESWT parameters. Forest plots were created to illustrate the combined effect estimates, and *P* values below 0.05 were set as of statistical significance.

## Results

### Study selection and characteristics

**Figure 1:**
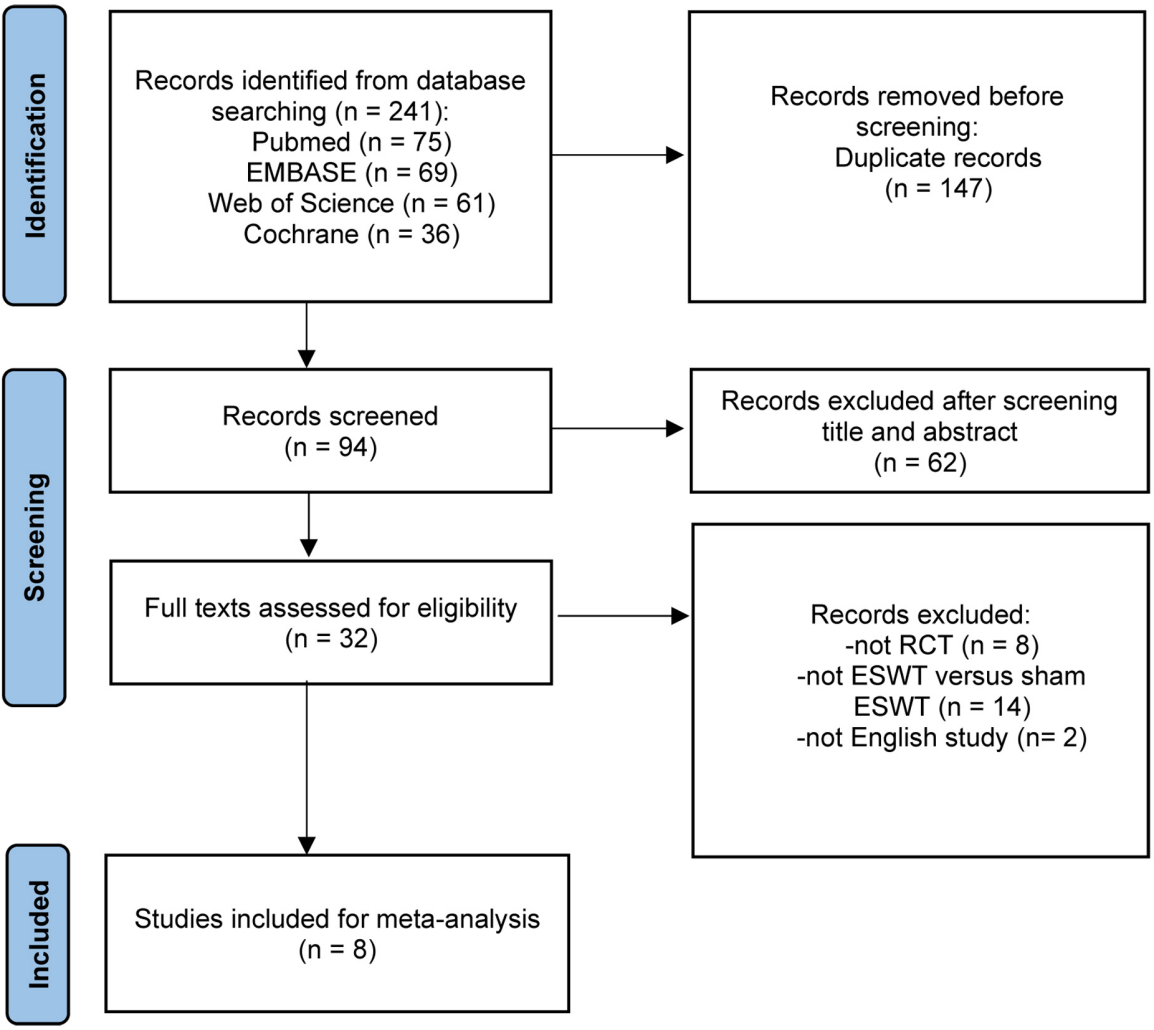
Flow chart of literature retrieval.

A search of PubMed, EMBASE, the Cochrane Library, and Web of Science identified 241 studies. After removing 147 duplicates, 94 records underwent title and abstract screening. Of these, 62 were excluded, leaving 32 studies for full-text review. After reviewing the full texts, 24 studies were excluded (eight were not RCTs, 14 did not compare ESWT *versus* sham ESWT, and two were non-English studies), resulting in eight studies (Abdelkader et al., 2021; Alsulaimani et al., 2025; Costa et al., 2005; Gatz et al., 2021; Mansur et al., 2021; Pinitkwamdee et al., 2020; Rasmussen et al., 2008; Vahdatpour et al., 2018) being included in the final meta-analysis (Fig. 1).

### Characteristics of the included studies

The included studies were conducted between 2005 and 2024 across various countries, including the UK, Denmark, Iran, Thailand, Egypt, Germany, Brazil, and Australia. A total of 458 participants were involved, with sample sizes ranging from 31 to 119 per study. The mean age of participants ranged from 28.3 to 61.4 years, and symptom duration varied from a minimum 3 months to a mean of 76.2 months (Table 1).

Four studies used rESWT, three studies used fESWT, and one study used a combination of rESWT and fESWT, with protocols typically consisting of three to four sessions over 4 to 6 weeks, delivering 1,500 to 4,500 shocks per session at intensities of 0.1 to 0.6 mJ/mm^2^ or 1.5 to 5 bars. Sham ESWT protocols were designed to mimic the real treatment, using methods such as zero-energy settings, disconnected probes, air-filled cavities, or interposed materials to dissipate waves while preserving sounds, positioning, and tactile cues. Seven studies applied double-blind design (patients and elevators), while only one study applied single-blind design (patients), with three studies reported excellent blind validation. Most studies incorporated co-interventions such as eccentric exercises, stretching, or education in both ESWT and sham ESWT groups, ensuring consistency across treatment arms, though specific regimens varied (Table 2).

### Risk of bias

**Figure 2:**
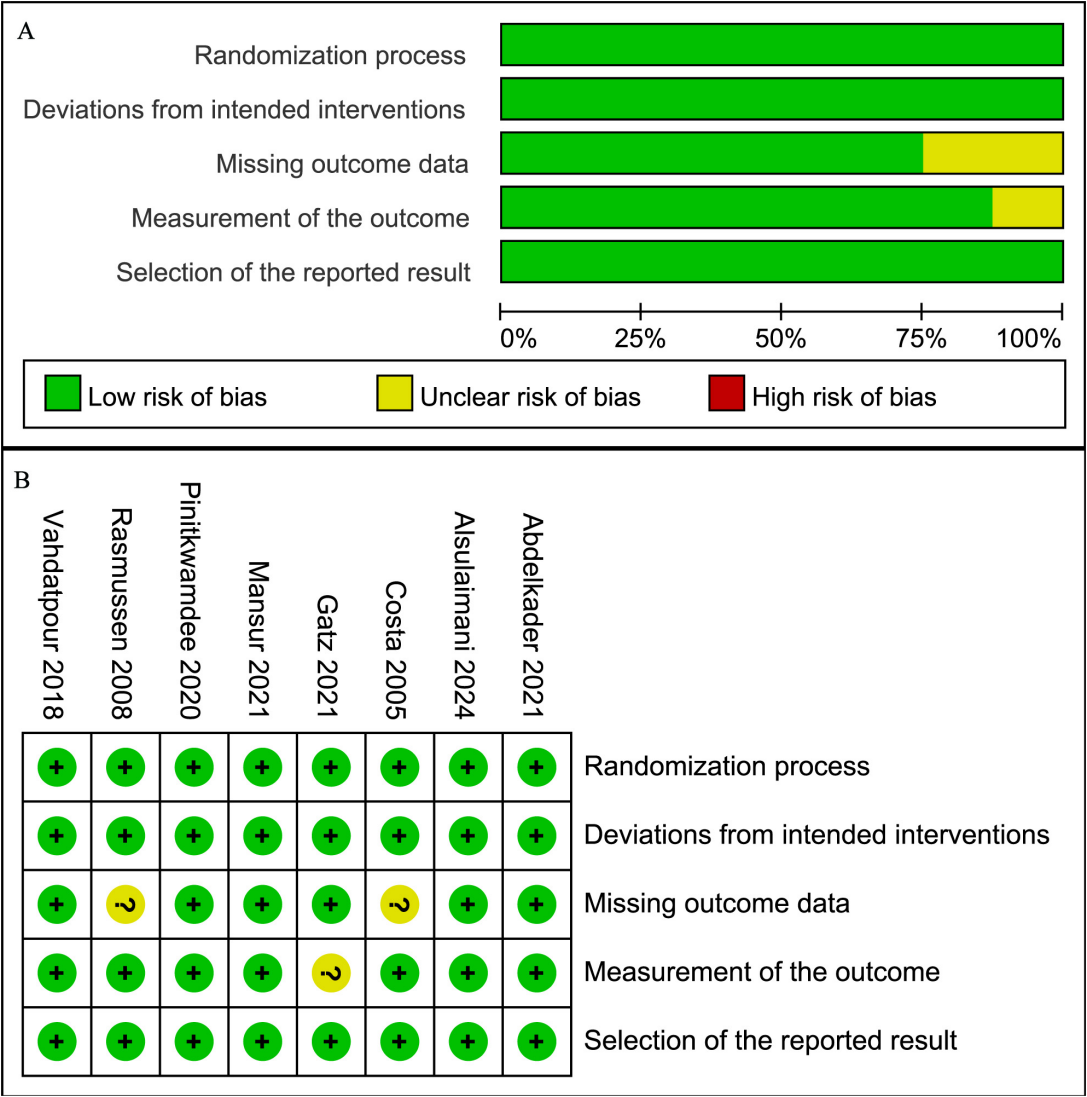
Risk of bias graph. (A) Graph of the risk of bias summary for the included studies; (B) Graph of the risk of bias for each included study (Abdelkader et al., 2021; Alsulaimani et al., 2025; Costa et al., 2005; Gatz et al., 2021; Mansur et al., 2021; Pinitkwamdee et al., 2020; Rasmussen et al., 2008; Vahdatpour et al., 2018).

The methodological quality of the included studies was assessed using the RoB 2. Most studies showed a low risk of bias across the five domains, while some studies had an unclear risk in measurement of the outcome and missing outcome data. Specifically, two RCTs (Costa et al., 2005; Rasmussen et al., 2008) had an unclear risk in missing outcome data due to incomplete follow-up reporting, and one RCT (Gatz et al., 2021) had an unclear risk in measurement of the outcome due to patient-only blinding. The remaining RCTs were rated as low risk across all domains (Fig. 2).

**Table 1:** Characteristics of the included studies.

First author	Year	Country	Sample size, *n*		Age, years Mean ± SD		Sex (M/F)		Symptom duration, months Mean ± SD (range)	
			ESWT	Placebo	ESWT	Placebo	ESWT	Placebo	ESWT	Placebo
Costa	2005	UK	22	27	58.7 ± 10.8	47.7 ± 13.5	9/13	12/15	17.8 ± 10.1	20.8 ± 21.2
Rasmussen	2008	Denmark	24	24	49 ± 9	46 ± 13	12/12	8/16	3-12	
Vahdatpour	2018	Iran	22	21	54.9 ± 11.3	54.3 ± 12.4	4/18	4/17	4.32 ± 1.55	4.51 ± 1.87
Pinitkwamdee	2020	Thailand	16	15	61.4 ± 5.9	56.5 ± 7.9	2/14	5/10	7.5 ± −(6–120)	12 ± −(6–48)
Abdelkader	2021	Egypt	25	25	29.9 ± 7.0	28.3 ± 6.8	22/28		6–12	
Gatz	2021	Germany	21	21	43 ± 13.31	51 ± 10.36	15/6	13/8	24 ± 21.70	21 ± 23.79
Mansur	2021	Brazil	58	61	52.2 ± 10.9	53.5 ± 11.4	31/27	30/31	66.0 ± 96.7	76.2 ± 98.2
Alsulaimani	2025	Australia	38	38	48.5 ± 12.2	52.5 ± 11.1	19/19	17/21	33.3 ± 44.1	47.8 ± 71

**Notes.**

M male F female NR not reported ESWT extracorporeal shockwave therapy UK the United Kingdom fESWT focused extracorporeal shockwave therapy rESWT radial extracorporeal shockwave therapy

**Table 2:** Details of ESWT and control group.

First author (Year)	ESWT type	Frequency (Hz)	Intensity (Energy)	Total shocks	Sessions (Interval, Duration)	Sham description	Blinding	Co-interventions
Costa et al. (2005)	fESWT –Electromagnetic, Storz Modulith SLK (4 mm focal area, US-guided)	NR	0.2 mJ/mm^2^	1,500	3 (1/month, 3 months)	Bubble wrap (opaque cloth) interposed for air gap; dissipates waves but mimics sounds/settings/positioning.	Double-blind (patients/assessors); no validation.	None during trial
Rasmussen et al. (2008)	rESWT - Piezoson 100 (to swelling/tenderness; no US)	50	0.12–0.51 mJ/mm^2^	2,000	4 (1/week, 4 weeks)	Same machine/probe/positioning/sounds at zero energy.	Double-blind (patients/evaluator); no validation.	Stretching + eccentric training (4 weeks).
Vahdatpour et al. (2018)	fESWT + rESWT - Duolith SD, Storz (reposition every 500 shocks)	3 (fESWT), 21 (rESWT)	0.25–0.4 mJ/mm^2^ (fESWT), 1.8-2.6 mJ/mm^2^ (rESWT)	1,500 (fESWT) + 3,000 (rESWT)	4 (1/week, 4 weeks)	Same positioning/machine sounds/probe movements at zero energy.	Double-blind (patients/evaluator); no validation.	Calf stretching + massage + eccentric training (4 weeks); diclofenac 100 mg/d (2 weeks).
Pinitkwamdee et al. (2020)	rESWT - Swiss DolorClast Classic (15 mm probe to tenderness ≤2 cm from insertion)	8–12	0.12–0.16 mJ/mm^2^	2,000	4 (1/week, 4 weeks)	Same blue radial probe/gel/settings/sounds but disconnected (red non-contact probe for sound only, no waves).	Double-blind (patients/assessor); validation: <50% correct guessing.	Avoid co-interventions/pain-provoking activities; rescue meds (naproxen 750 mg/d or acetaminophen 2,000–4,000 mg/d).
Abdelkader et al. (2021)	fESWT - DOULITH SD1, Storz (circumferential to max tenderness)	8	0.1 mJ/mm^2^	2,000	4 (1/week, 4 weeks)	Gel applied/handpiece moved identically at zero energy (same sounds/positioning).	Double-blind (patients/independent assessor); no validation.	Eccentric calf training (3 ×15 reps 2 ×/d, 7 d/wk, 4 weeks); stretching (3 ×30 s 2 ×/d, 7 d/wk, 4 weeks); avoid NSAIDs.
Gatz et al. (2021)	fESWT - Piezowave 2, Richard Wolf (gel pad; sliding on painful area; no US)	5	Mean 0.6 mJ/mm^2^	2,000 (+500 to calf)	4 (every 2 weeks, 6 weeks)	Same settings/sounds/positioning/gel pad but air-filled cavity blocks waves.	Single-blind (patients only); no validation.	Daily physiotherapy (24 weeks: eccentric 3 × 152 ×/d; stretching 1 ×/d; isometric 5 reps 1 ×/d); no sports (first 6 weeks).
Mansur et al. (2021)	rESWT - BLT600, BTL (to bulge/crease; prone)	7–10	1.5–2.5 bars	2000-3000	3 (every 2 weeks, 4 weeks)	Identical settings/positioning/protocol but firing transmission piece removed (no waves, same sounds).	Double-blind (patients/providers); validation: 85% patients/70% providers incorrect.	Eccentric exercises (modified Alfredson: 3 × 152 ×/d, 7 d/wk, 3 months); no sports (8 weeks).
Alsulaimani et al. (2025)	rESWT - Intellect RPW2, Chattanooga (to 3–4 cm^2^ painful insertion)	10	2–5 bars	3,000	3 (5–10 days apart, 10–20 days)	Identical device/noise/positioning/gel but pressure-minimizing component removed (no propagation, same auditory/tactile cues).	Double-blind (patients/independent assessor); validation: 81% patients incorrect.	Education; progressive isometric/eccentric calf raises (4 ×15, knee straight/bent, 3 ×/wk); paracetamol ≤4 g/d if needed; no other treatments/NSAIDs.

**Notes.**

NR not reported ESWT extracorporeal shockwave therapy fESWT focused extracorporeal shockwave therapy rESWT radial extracorporeal shockwave therapy US ultrasound NSAID non-steroidal anti-inflammatory drugs

### VAS

**Figure 3:**
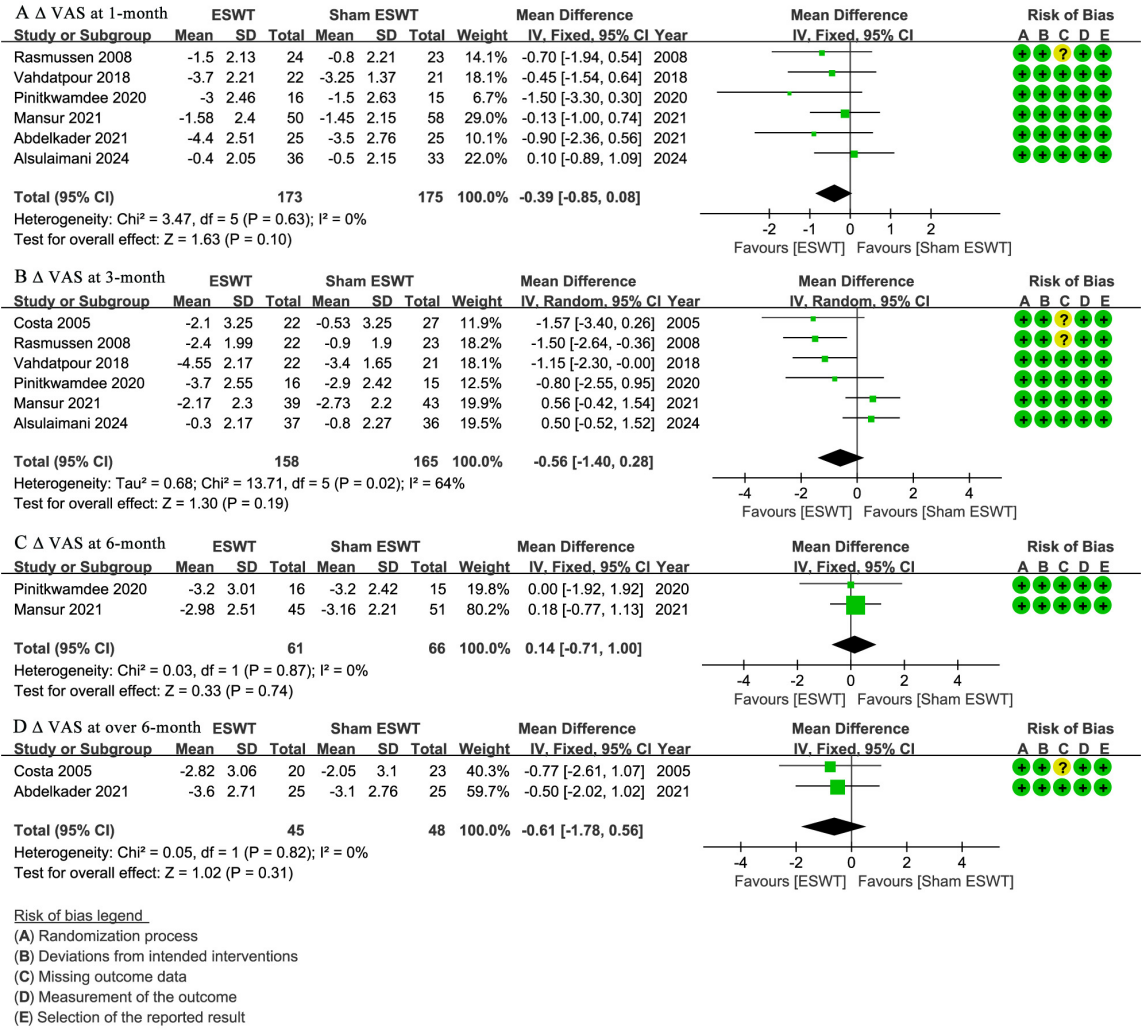
Meta-analysis of ΔVAS at (A) 1-month follow-up, (B) 3-month follow-up, (C) 6-month follow-up, and (D) over 6-month follow-up. The green squares represent the effect estimate of the individual studies, the horizontal lines indicate the confidence interval, and the dimension of the square reflects the weight of each study. The black diamond represents the combined point estimate and confidence intervals. (VAS, Visual Analog Scale) (Rasmussen et al., 2008; Vahdatpour et al., 2018; Pinitkwamdee et al., 2020; Mansur et al., 2021; Abdelkader et al., 2021; Alsulaimani et al., 2025; Costa et al., 2005).

At 1-month follow-up, six studies (Abdelkader et al., 2021; Alsulaimani et al., 2025; Mansur et al., 2021; Pinitkwamdee et al., 2020; Rasmussen et al., 2008; Vahdatpour et al., 2018) reported ΔVAS, showing no significant difference between the ESWT and sham ESWT groups (WMD, −0.39; 95% CI [−0.85 to 0.08]; I^2^ = 0%; *P* = 0.10) (Fig. 3A).

At 3-month follow-up, six studies (Alsulaimani et al., 2025; Costa et al., 2005; Mansur et al., 2021; Pinitkwamdee et al., 2020; Rasmussen et al., 2008; Vahdatpour et al., 2018) reported ΔVAS, showing no significant difference between the groups, with a non-significant trend favoring ESWT (WMD, −0.56; 95% CI [−1.40 to 0.28]; I^2^ = 64%; *P* = 0.19) (Fig. 3B).

At 6-month follow-up, two studies (Mansur et al., 2021; Pinitkwamdee et al., 2020) reported ΔVAS, showing no significant difference between the ESWT and sham ESWT groups (WMD, 0.14; 95% CI [−0.71 to 1.00]; I^2^ = 0%; *P* = 0.74) (Fig. 3C).

At over 6-month follow-up, two studies (Abdelkader et al., 2021; Costa et al., 2005) reported ΔVAS, showing no significant difference between the groups, with a non-significant trend favoring ESWT (WMD, −0.61; 95% CI [−1.78 to 0.56]; I^2^ = 0%; *P* = 0.31) (Fig. 3D).

### VISA-A

**Figure 4:**
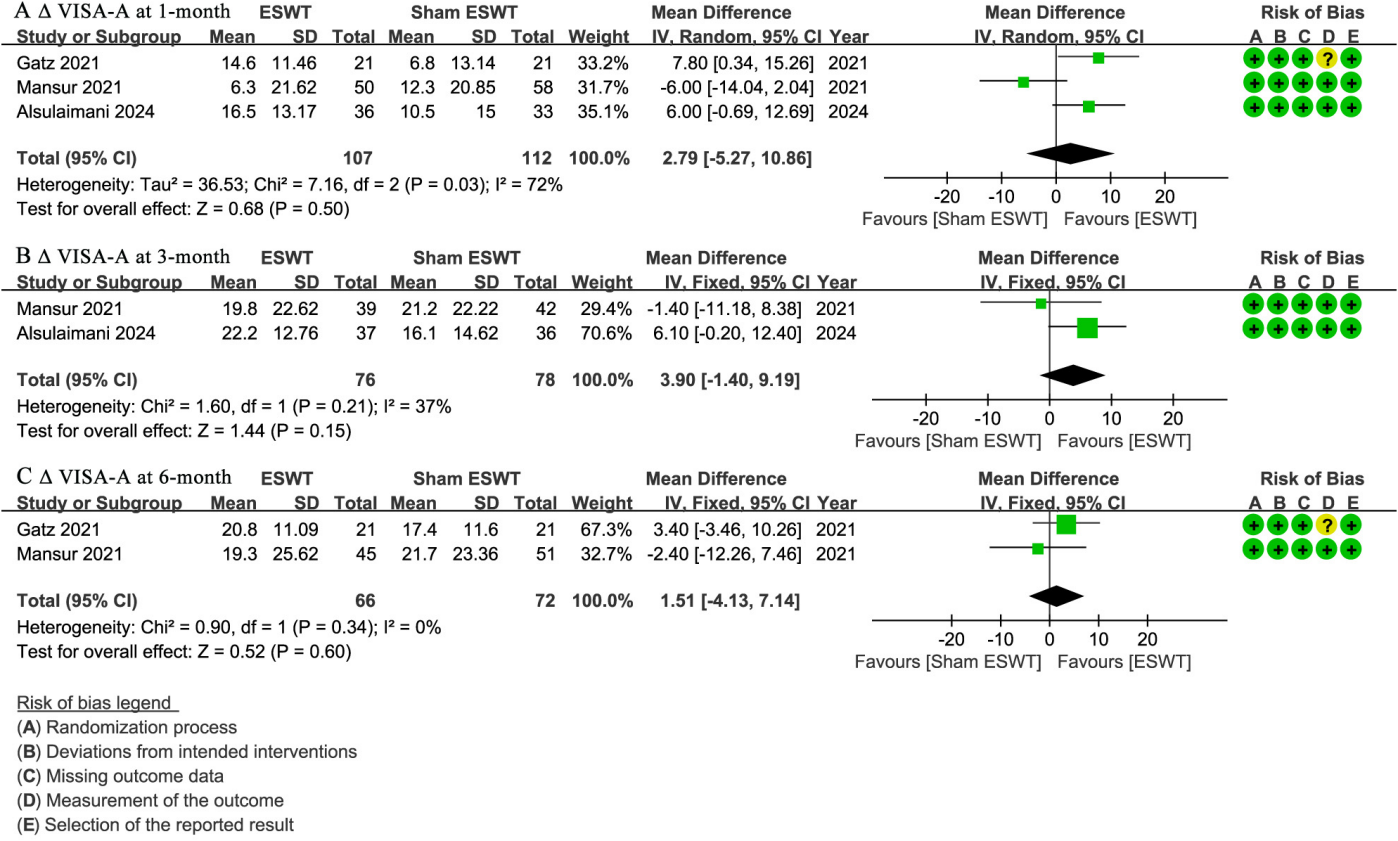
Meta-analysis of ΔVISA-A at (A) 1-month follow-up, (B) 3-month follow-up, and (C) 6-month follow-up. The green squares represent the effect estimate of the individual studies, the horizontal lines indicate the confidence interval, and the dimension of the square reflects the weight of each study. The black diamond represents the combined point estimate and confidence intervals. (VISA-A, Victorian Institute of Sports Assessment-Achilles questionnaire) (Gatz et al., 2021; Mansur et al., 2021; Alsulaimani et al., 2025).

At 1-month follow-up, three studies (Alsulaimani et al., 2025; Gatz et al., 2021; Mansur et al., 2021) reported ΔVISA-A scores, showing no significant difference between the ESWT and sham ESWT groups, with a non-significant trend favoring ESWT (WMD, 2.79; 95% CI [−5.27 to 10.86]; I^2^ = 72%; *P* = 0.50) (Fig. 4A).

At 3-month follow-up, two studies (Alsulaimani et al., 2025; Mansur et al., 2021) reported Δ VISA-A scores, showing no significant difference between the groups, with a non-significant trend favoring ESWT (WMD, 3.90; 95% CI [−1.40 to 9.19]; I^2^ = 37%; *P* = 0.15) (Fig. 4B).

At 6-month follow-up, two studies (Gatz et al., 2021; Mansur et al., 2021) reported ΔVISA-A scores, showing no significant difference between the ESWT and sham ESWT groups (WMD, 1.51; 95% CI [−4.13 to 7.14]; I^2^ = 0%; *P* = 0.60) (Fig. 4C).

### AOFAS

**Figure 5:**
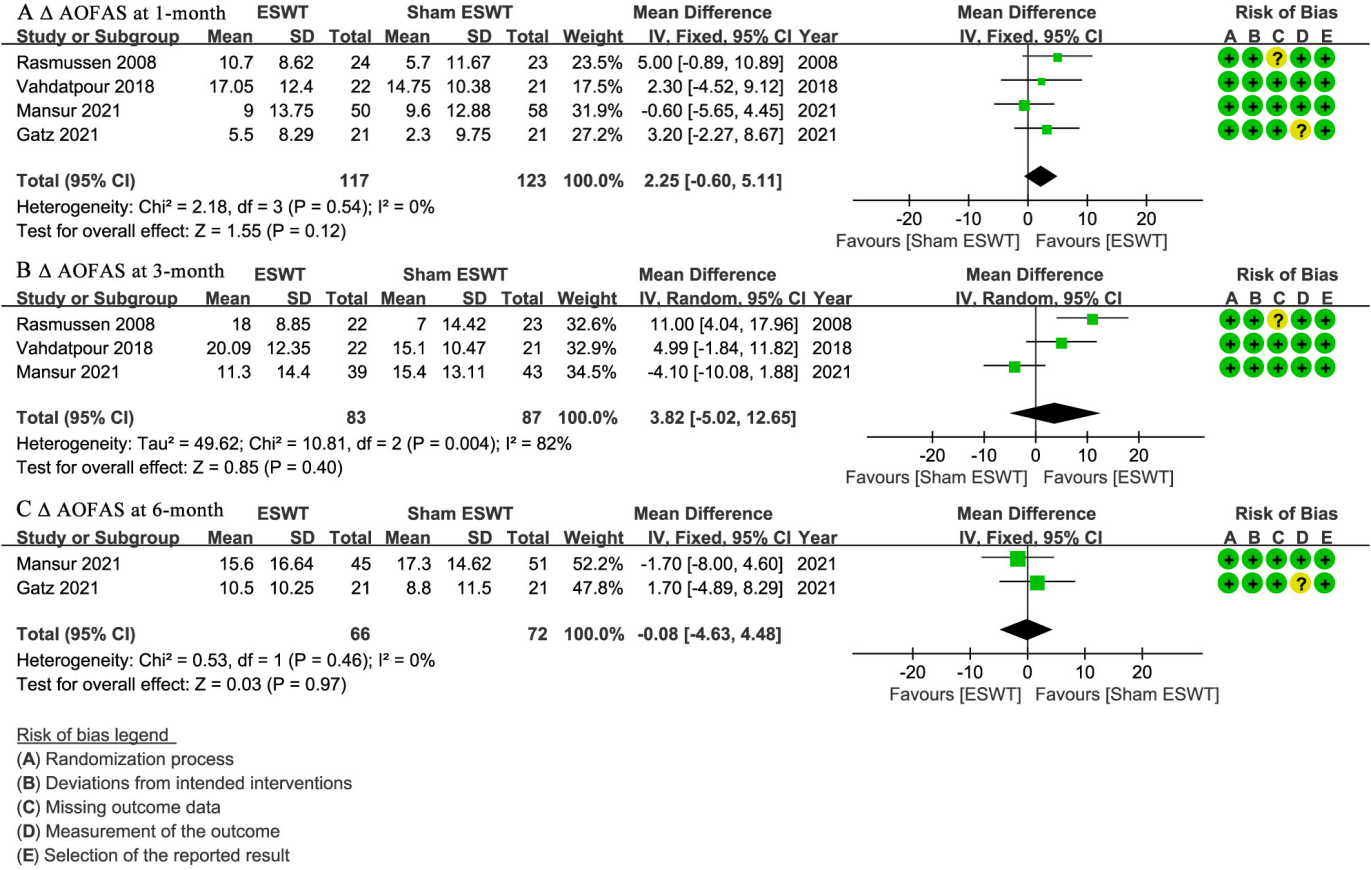
Meta-analysis of ΔAOFAS at (A) 1-month follow-up, (B) 3-month follow-up, and (C) 6-month follow-up. The green squares represent the effect estimate of the individual studies, the horizontal lines indicate the confidence interval, and the dimension of the square reflects the weight of each study. The black diamond represents the combined point estimate and confidence intervals. (AOFAS, American Orthopaedic Foot and Ankle Society) (Rasmussen et al., 2008; Vahdatpour et al., 2018; Mansur et al., 2021; Gatz et al., 2021).

At 1-month follow-up, four studies (Gatz et al., 2021; Mansur et al., 2021; Rasmussen et al., 2008; Vahdatpour et al., 2018) reported Δ AOFAS scores, showing no significant difference between the ESWT and sham ESWT groups (WMD, 2.25; 95% CI [−0.60 to 5.11]; I^2^ = 0%; *P* = 0.12) (Fig. 5A).

At 3-month follow-up, three studies (Mansur et al., 2021; Rasmussen et al., 2008; Vahdatpour et al., 2018) reported Δ AOFAS scores, showing no significant difference between the groups, with a non-significant trend favoring ESWT (WMD, 3.82; 95% CI [−5.02 to 12.65]; I^2^ = 82%; *P* = 0.40) (Fig. 5B).

At 6-month follow-up, two studies (Gatz et al., 2021; Mansur et al., 2021) reported Δ AOFAS scores, showing no significant difference between the ESWT and sham ESWT groups (WMD, −0.08; 95% CI [−4.63 to 4.48]; I^2^ = 0%; *P* = 0.97) (Fig. 5C).

### Adverse events

**Figure 6:**
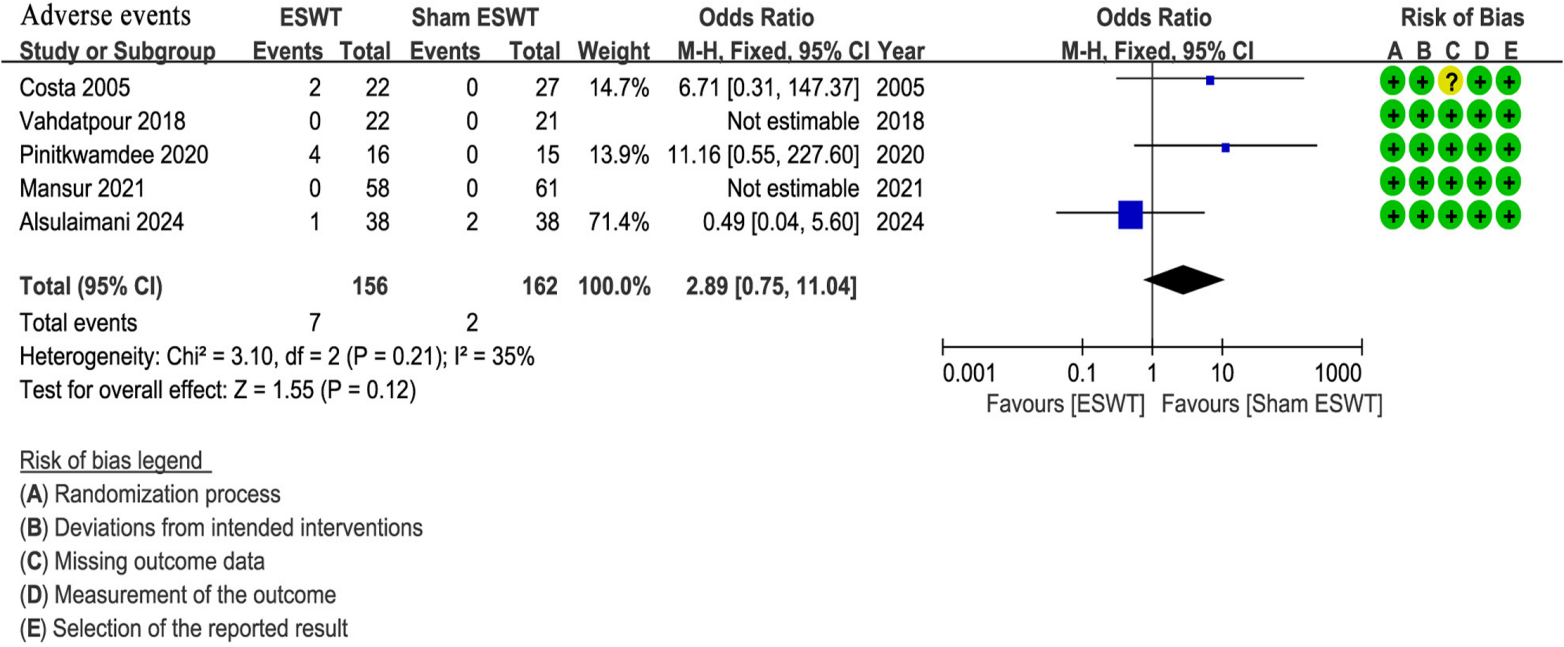
Meta-analysis of adverse events. The blue squares represent the effect estimate of the individual studies, the horizontal lines indicate the confidence interval, and the dimension of the square reflects the weight of each study. The black diamond represents the combined point estimate and confidence intervals (Costa et al., 2005; Vahdatpour et al., 2018; Pinitkwamdee et al., 2020; Mansur et al., 2021; Alsulaimani et al., 2025).

Five studies (Alsulaimani et al., 2025; Costa et al., 2005; Mansur et al., 2021; Pinitkwamdee et al., 2020; Vahdatpour et al., 2018) reported adverse events, all of which were mild and did not require specific medical intervention. The ESWT group showed a higher incidence of adverse events (4.5%, 7/156) compared to the sham ESWT group (1.2%, 2/162). However, the difference between the groups was not statistically significant (OR, 2.89; 95% CI [0.75–11.04]; I^2^ = 35%; *P* = 0.12) (Fig. 6).

### Summary of primary findings with GRADE and sensitivity analyses

The GRADE methodology was used to assess the certainty of evidence for each outcome (Table 3). The quality of evidence was moderate for ΔVAS at 1-month, ΔVAS at 6-month, ΔVISA-A at 3-month, ΔAOFAS at 1-month, and adverse events, and low for the remaining outcomes (Table 3).

**Table 3:** GRADE summary of findings.

**ESWT for** ** chronic Achilles tendinopathy**				
**Patient or population:** Adult patients with chronic Achilles tendinopathy **Intervention:** ESWT **Comparison:** Sham ESWT				

**Outcomes**	**Effect estimate**	**95% CI**	**No of participants (studies)**	**Quality of the evidence** **(GRADE)**
**ΔVAS at 1-month**	WMD: 0.39 lower in the interventional group.	0.85 lower to 0.08 higher	348 (6 studies)	⊕⊕⊕⊝**moderate**
**ΔVAS at 3-month**	WMD: 0.56 lower in the interventional group.	1.40 lower to 0.28 higher	323 (6 studies)	⊕⊕⊝⊝**low**
**ΔVAS at 6-month**	WMD: 0.14 higher in the interventional group.	0.71 lower to 1.00 higher	127 (2 studies)	⊕⊕⊕⊝**moderate**
**ΔVAS at over 6-month**	WMD: 0.61 lower in the interventional group.	1.78 lower to 0.56 higher	93 (2 studies)	⊕⊕⊝⊝**low**
**ΔVISA-A at 1-month**	WMD: 2.79 higher in the interventional group.	5.27 lower to 10.86 higher	219 (3 studies)	⊕⊕⊝⊝**low**
**ΔVISA-A at 3-month**	WMD: 3.90 higher in the interventional group.	1.40 lower to 9.19 higher	154 (2 studies)	⊕⊕⊕⊝**moderate**
**ΔVISA-A at 6-month**	WMD: 1.51 higher in the interventional group.	4.13 lower to 7.14 higher	138 (2 studies)	⊕⊕⊝⊝**low**
**ΔAOFAS at 1-month**	WMD: 2.25 higher in the interventional group.	0.60 lower to 5.11 higher	240 (4 studies)	⊕⊕⊕⊝**moderate**
**ΔAOFAS at 3-month**	WMD: 3.82 higher in the interventional group.	5.02 lower to 12.65 higher	170 (3 studies)	⊕⊕⊝⊝**low**
**ΔAOFAS at 6-month**	WMD: 0.08 lower in the interventional group.	4.63 lower to 4.48 higher	138 (2 studies)	⊕⊕⊝⊝**low**
**Adverse events**	OR: 2.89 higher in the interventional group.	0.75 lower to 11.04 higher	318 (5 studies)	⊕⊕⊕⊝**moderate**
**GRADE:** Grading of Recommendations, Assessment, Development and Evaluation; ** WMD:** Weighted mean differences; ** OR:** Odds ratios; ** CI:** Confidence interval; ** ESWT:** Extracorporeal shockwave therapy; ** VAS:** Visual Analog Scale; ** VISA-A:** Victorian Institute of Sports Assessment-Achilles questionnaire; ** AOFAS:** American Orthopaedic Foot and Ankle Society.				
GRADE Working Group grades of evidence **High quality:** Further research is very unlikely to change our confidence in the estimate of effect. **Moderate quality:** Further research is likely to have an important impact on our confidence in the estimate of effect and may change the estimate. **Low quality:** Further research is very likely to have an important impact on our confidence in the estimate of effect and is likely to change the estimate. **Very low quality:** We are very uncertain about the estimate.				

Sensitivity analyses were performed by excluding the studies with significantly different ESWT parameters. Specifically, the study by Costa et al. (2005), which used monthly sessions over 3 months with low-energy fESWT (0.2 mJ/mm^2^, 1,500 shocks per session), and the study by Vahdatpour et al. (2018), which combined focused and radial ESWT with a broader range of frequencies (three Hz for focused, 21 Hz for radial) and higher energies for the radial component (up to 2.6 mJ/mm^2^, totaling 4,500 shocks per session), were excluded. The sensitivity analyses showed no significant changes in pooled estimates, confidence intervals, or I^2^ values for any outcome, confirming the robustness of the primary findings.

### Hypothesis-generating findings from subgroup analyses

We conducted subgroup analyses to explore how the type of ESWT might influence outcomes. No statistically significant differences emerged across these outcomes or ESWT types, with all *P*-values exceeding 0.05 (Table 4).

**Table 4:** Subgroup analyses according to the ESWT type.

	No. of studies (No. of patients)	WMD or OR	95% CI	*I* ^2^	*P* value		Comparison
**ΔVAS at 1-month**						
Overall	6 (348)	−0.39	−0.85, 0.08	0%	0.10		ESWT = sham
rESWT	5 (305)	−0.37	−0.89, 0.14	0%	0.15		rESWT = sham
rESWT + fESWT	1 (43)	−0.45	−1.45, 0.64	NA	0.42		rESWT + fESWT= sham
**ΔVAS at 3-month**						
Overall	6 (323)	−0.56	−1.40, 0.28	64%	0.19		ESWT = sham
rESWT	231 (4)	−0.24	−1.28, 0.81	68%	0.65		rESWT = sham
fESWT	1 (49)	−1.57	−3.40, 0.26	NA	0.09		fESWT = sham
rESWT + fESWT	1 (43)	−1.15	−2.30, 0.02	NA	0.06		rESWT + fESWT= sham
**ΔVAS at 6-month**						
Overall	2 (127)	0.14	−0.71, 1.00	0%	0.74		ESWT = sham
rESWT	2 (127)	0.14	−0.71, 1.00	0%	0.74		fESWT = sham
**ΔVAS at over 6-month**						
Overall	2 (93)	−0.61	−1.78, 0.56	0%	0.31	ESWT = sham	
rESWT	1 (50)	−0.50	−2.02, 1.02	NA	0.52	rESWT = sham	
fESWT	1 (43)	−0.77	−2.61, 1.07	NA	0.41	fESWT = sham	
**ΔVISA-A score at 1-month**						
Overall	3 (219)	3.26	−0.98, 7.49	72%	0.13	ESWT = sham	
rESWT	2 (177)	0.22	−11.54, 11.97	80%	0.97	rESWT = sham	
fESWT	1 (42)	6.37	−1.22, 10.26	NA	0.12	fESWT = sham	
**ΔVISA-A score at 3-month**						
Overall	2 (154)	3.90	−1.40, 9.19	37%	0.15	ESWT = sham	
rESWT	2 (154)	3.90	−1.40, 9.19	37%	0.15	rESWT = sham	
**ΔVISA-A score at 6-month**						
Overall	2 (138)	1.51	−4.13, 7.14	0%	0.60	ESWT = sham	
rESWT	1 (96)	−2.40	−12.26, 7.46	NA	0.63	rESWT = sham	
fESWT	1 (42)	3.40	−3.46, 10.26	NA	0.33	fESWT = sham	
**ΔAOFAS score at 1-month**						
Overall	240 (4)	2.25	−0.60, 5.11	0%	0.12	ESWT = sham	
rESWT	155 (2)	1.99	−3.48, 7.46	50%	0.48	rESWT = sham	
fESWT	42 (1)	3.20	−2.27, 8.67	NA	0.25	fESWT = sham	
rESWT + fESWT	43 (1)	2.30	−4.52, 9.12	NA	0.51	rESWT + fESWT= sham	
**ΔAOFAS score at 3-month**						
Overall	170 (3)	3.82	−5.02, 12.65	82%	0.40	ESWT = sham	
rESWT	127 (2)	3.34	−11.46, 18.14	90%	0.66	rESWT = sham	
rESWT + fESWT	43 (1)	4.99	−1.84, 11.82	NA	0.15	rESWT + fESWT= sham	
**ΔAOFAS score at 6-month**						
Overall	138 (2)	−0.08	−4.63, 4.48	0%	0.97	ESWT = sham	
rESWT	96 (1)	−1.70	−8.00, 4.60	NA	0.60	rESWT = sham	
fESWT	42 (1)	1.70	−4.89, 8.29	NA	0.61	fESWT = sham	
**Adverse events**						
Overall	318 (5)	2.89	0.75, 11.04	35%	0.12	ESWT = sham	
rESWT	226 (3)	2.06	0.09, 46.09	61%	0.65	rESWT = sham	
fESWT	49 (1)	6.71	0.31, 147.37	NA	0.23	fESWT = sham	
rESWT + fESWT	43 (1)	Not estimate	Not estimate	NA	NA	NA	

**Notes.**

ESWT extracorporeal shockwave therapy rESWT radial extracorporeal shockwave therapy fESWT focused extracorporeal shockwave therapy WMD weighted mean difference OR odds ratio CI confidence interval VAS visual analogue scale VISA-A the Victorian Institute of Sports Assessment-Achilles questionnaire AOFAS American Orthopaedic Foot and Ankle Society score NA not applicable

We then investigated whether the type of Achilles tendinopathy, classified as insertional, non-insertional, or mixed, affected outcomes. Similarly, no significant differences were observed between ESWT and sham ESWT in any subgroup, with all *P*-values greater than 0.05 (Table 5).

**Table 5:** Subgroup analyses according to the Achilles tendinopathy type.

	No. of studies (No. of patients)	WMD or OR			95% CI	*I* ^2^	*P* value			Comparison
**ΔVAS at 1-month**						
Overall		6 (348)	−0.39			−0.85, 0.08			0%	0.10			ESWT = sham
Insertional		3 (208)	−0.20			−0.82, 0.41			16%	0.52			ESWT = sham
Non-Insertional		1 (50)	−0.90			−2.36, 0.56			NA	0.23			ESWT = sham
Mixed		2 (90)	−0.56			−1.38, 0.26			0%	0.18			ESWT = sham
**ΔVAS at 3-month**						
Overall		6 (323)	−0.56			−1.40, 0.28			64%	0.19			ESWT = sham
Insertional		186 (3)	0.35			−0.31, 1.00	0%	0.30			ESWT = sham
Mixed		137 (3)	−1.37			−2.11, 0.63			0%	0.08			ESWT = sham
**ΔVAS at 6-month**						
Overall		2 (127)	0.14			−0.71, 1.00	0%	0.74			ESWT = sham
Insertional		2 (127)	0.14			−0.71, 1.00	0%	0.74			ESWT = sham
**ΔVAS at over 6-month**						
Overall	2 (93)			−0.61	−1.78, 0.56		0%		0.31	ESWT = sham	
Non-Insertional	1 (50)			−0.50	−2.02, 1.02		NA		0.52	ESWT = sham	
Mixed	1 (43)			−0.77	−2.61, 1.07		NA		0.41	ESWT = sham	
**ΔVISA-A score at 1-month**						
Overall	3 (219)			3.26	−0.98, 7.49		72%		0.13	ESWT = sham	
Insertional	2 (177)			0.22	−11.54, 11.97		80%		0.97	ESWT = sham	
Mixed	1 (42)			6.37	−1.22, 10.26		NA		0.12	ESWT = sham	
**ΔVISA-A score at 3-month**						
Overall	2 (154)			3.90	−1.40, 9.19		37%		0.15	ESWT = sham	
Insertional	2 (154)			3.90	−1.40, 9.19		37%		0.15	ESWT = sham	
**ΔVISA-A score at 6-month**						
Overall	2 (138)			1.51	−4.13, 7.14		0%		0.60	ESWT = sham	
Insertional	1 (96)			−2.40	−12.26, 7.46		NA		0.63	ESWT = sham	
Mixed	1 (42)			3.40	−3.46, 10.26		NA		0.33	ESWT = sham	
**ΔAOFAS score at 1-month**						
Overall	240 (4)			2.25	−0.60, 5.11		0%		0.12	ESWT = sham	
Insertional	108 (1)			−0.60	−5.65, 4.45		NA		0.82	ESWT = sham	
Mixed	132 (3)			3.49	−0.27, 7.05		0%		0.07	ESWT = sham	
**ΔAOFAS score at 3-month**						
Overall	170 (3)			3.82	−5.02, 12.65		82%		0.40	ESWT = sham	
Insertional	82 (1)			−4.10	−10.08, 1.88		NA		0.18	ESWT = sham	
Mixed	88 (2)			3.96	−2.14, 7.85		31%		0.08	ESWT = sham	
**ΔAOFAS score at 6-month**						
Overall	138 (2)			−0.08	−4.63, 4.48		0%		0.97	ESWT = sham	
Insertional	96 (1)			−1.70	−8.00, 4.60		NA		0.60	ESWT = sham	
Mixed	42 (1)			1.70	−4.89, 8.29		NA		0.61	ESWT = sham	
**Adverse events**						
Overall	318 (5)			2.89	0.75, 11.04		35%		0.12	ESWT = sham	
Insertional	226 (3)			2.06	0.09, 46.09		61%		0.65	ESWT = sham	
Mixed	92 (2)			6.71	0.31, 147.37		NA		0.23	ESWT = sham	

**Notes.**

ESWT extracorporeal shockwave therapy rESWT radial extracorporeal shockwave therapy fESWT focused extracorporeal shockwave therapy WMD weighted mean difference OR odds ratio CI confidence interval VAS visual analogue scale VISA-A the Victorian Institute of Sports Assessment-Achilles questionnaire AOFAS American Orthopaedic Foot and Ankle Society score NA not applicable

Finally, we analyzed outcomes based on symptom duration, dividing patients into those with symptoms lasting less than 12 months and those with symptoms for 12 months or longer. For patients with symptoms under 12 months, ESWT significantly improved ΔVAS compared to sham ESWT at 1-month (WMD, −0.76; 95% CI [−1.42 to −0.09]; I^2^ = 0%; *P* = 0.03) and 3-month follow-ups (WMD, −1.23; 95% CI [−1.97 to −0.50]; I^2^ = 0%; *P* = 0.001). However, no significant differences were found for other outcomes or for patients with symptoms lasting 12 months or more, with all *P*-values above 0.05 (Table 6).

**Table 6:** Subgroup analyses according to the mean symptom duration.

	No. of studies (No. of patients)	WMD or OR	95% CI	*I* ^2^		*P* value		Comparison	
**ΔVAS at 1-month**						
Overall	6 (348)	−0.39	−0.85, 0.08	0%		0.10		ESWT = sham	
<12 months	4 (173)	−0.76	−1.42, −0.09	0%		0.03		ESWT > sham	
≥12 months	2 (177)	−0.03	−0.68, 0.62	0%		0.93		ESWT = sham	
**ΔVAS at 3-month**						
Overall	6 (323)	−0.56	−1.40, 0.28	64%		0.19		ESWT = sham	
<12 months	3 (119)	−1.23	−1.97, −0.50	0%		0.001		ESWT > sham	
≥12 months	3 (204)	0.09	−0.95, 1.12	55%		0.87		ESWT = sham	
**ΔVAS at 6-month**						
Overall	2 (127)	0.14	−0.71, 1.00	0%		0.74		ESWT = sham	
<12 months	1 (31)	0.00	−1.92, 1.92	NA		1.00		ESWT = sham	
≥12 months	1 (96)	0.18	−0.77, 1.13	NA		0.71		ESWT = sham	
**ΔVAS at over 6-month**						
Overall	2 (93)	−0.61	−1.78, 0.56		0%		0.31		ESWT = sham
<12 months	1 (50)	−0.50	−2.02, 1.02		NA		0.52		ESWT = sham
≥12 months	1 (43)	−0.77	−2.61, 1.07		NA		0.41		ESWT = sham
**ΔVISA-A score at 1-month**						
Overall	3 (219)	3.26	−0.98, 7.49		72%		0.13		ESWT = sham
≥12 months	3 (219)	3.26	−0.98, 7.49		72%		0.13		ESWT = sham
**ΔVISA-A score at 3-month**						
Overall	2 (154)	3.90	−1.40, 9.19		37%		0.15		ESWT = sham
≥12 months	2 (154)	3.90	−1.40, 9.19		37%		0.15		ESWT = sham
**ΔVISA-A score at 6-month**						
Overall	2 (138)	1.51	−4.13, 7.14		0%		0.60		ESWT = sham
≥12 months	2 (138)	1.51	−4.13, 7.14		0%		0.60		ESWT = sham
**ΔAOFAS score at 1-month**						
Overall	240 (4)	2.25	−0.60, 5.11		0%		0.12		ESWT = sham
<12 months	90 (2)	3.85	−0.61, 8.30		0%		0.09		ESWT = sham
≥12 months	150 (2)	1.15	−2.56, 4.86		0%		0.54		ESWT = sham
**ΔAOFAS score at 3-month**						
Overall	170 (3)	3.82	−5.02, 12.65		82%		0.40		ESWT = sham
<12 months	88 (2)	3.96	−2.14, 7.85		31%		0.08		ESWT = sham
≥12 months	82 (1)	−4.10	−10.08, 1.88		NA		0.18		ESWT = sham
**ΔAOFAS score at 6-month**						
Overall	138 (2)	−0.08	−4.63, 4.48		0%		0.97		ESWT = sham
≥12 months	138 (2)	−0.08	−4.63, 4.48		0%		0.97		ESWT = sham
**Adverse events**						
Overall	318 (5)	2.89	0.75, 11.04		35%		0.12		ESWT = sham
<12 months	74 (2)	11.16	0.55, 227.60		NA		0.12		ESWT = sham
≥12 months	244 (3)	1.52	0.12, 19.73		42%		0.75		ESWT = sham

**Notes.**

ESWT extracorporeal shockwave therapy rESWT radial extracorporeal shockwave therapy fESWT focused extracorporeal shockwave therapy WMD weighted mean difference OR odds ratio CI confidence interval VAS visual analogue scale VISA-A the Victorian Institute of Sports Assessment-Achilles questionnaire AOFAS American Orthopaedic Foot and Ankle Society score NA not applicable

## Discussion

This meta-analysis included eight RCTs involving 458 patients comparing ESWT to sham ESWT for chronic Achilles tendinopathy. Primary findings, supported by low- to moderate-quality evidence, indicate that ESWT did not demonstrate superiority over sham ESWT. Pain reduction (ΔVAS) and functional improvement (ΔVISA-A and ΔAOFAS) revealed no significant differences at any follow-up. Although the ESWT group had a higher incidence of mild adverse events (4.5% *versus* 1.2% in the sham group), this difference was not statistically significant. Hypothesis-generating subgroup analyses based on ESWT type, tendinopathy classification, and symptom duration revealed statistically significant pain improvements only in patients with symptom duration <12 months, with sub-MCID improvement at 1-month (WMD: −0.76) and a clinically meaningful reduction at 3-months (WMD: −1.23) that exceeded the VAS MCID of 1.1 out of 10. Importantly, this benefit is transient and not robust, as it stems from exploratory subgroup analyses.

Previous systematic reviews and meta-analyses evaluating ESWT for Achilles tendinopathy have yielded varying conclusions (Charles et al., 2023; Fan et al., 2020; Feeney, 2022; Paantjens et al., 2022; Stania et al., 2019). A meta-analysis by Fan et al. (2020) reported that ESWT improves pain and function effectively, but included diverse control groups and non-RCTs, introducing heterogeneity and bias. Charles et al. (2023), Stania et al. (2019), and Paantjens et al. (2022) conducted broader reviews with mixed conclusions, but their heterogeneous control groups hindered the isolation of ESWT’s specific effectiveness. Feeney (2022) provided a narrative synthesis, concluding ESWT is at least as effective as other treatments, but lacking quantitative analysis and structured quality assessment.

Our study represents the first meta-analysis to exclusively compare ESWT with sham ESWT in chronic Achilles tendinopathy, thus isolating its specific effect by ensuring both groups received identical concomitant treatments. This rigorous approach across eight RCTs enhances evidence quality and reduces bias from variable co-interventions. Additionally, we evaluated multiple outcomes across structured time points, providing a more thorough analysis than prior studies. While previous reviews broadly concluded ESWT is effective for Achilles tendinopathy, our results suggest a more limited benefit, restricted to specific patient subsets.

Although our primary findings revealed no statistically significant differences overall, hypothesis-generating subgroup analyses indicated a transient benefit of ESWT in reducing pain (ΔVAS) among patients with symptom durations < 12 months. This included a statistically significant but subthreshold improvement at 1 month (WMD: −0.76), which fell short of the MCID of 1.1 points. In contrast, a clinically meaningful reduction at 3 months (WMD: −1.23) exceeded this threshold, suggesting a potential optimal early intervention window (Van der Worp et al., 2013). This temporal pattern likely reflects ESWT’s dose-dependent mechanisms (Abbas, Khan & Veqar, 2023; Ke et al., 2016; Yang et al., 2025), where mechanotransduction initiates healing through initial cellular responses and tissue repair, with sustained improvements in collagen remodeling developing over 1–3 months (Auersperg & Trieb, 2020; De la Corte-Rodríguez et al., 2023). Clinically, such early pain relief may enhance patient adherence without extended downtime, and reassessments at 3 months are recommended for subacute cases to capitalize on the tendon’s residual healing capacity, bridging reactive or dysrepair phases before degenerative progression (Majidi et al., 2024; Ryskalin et al., 2022; Simplicio et al., 2020). In contrast, diminished responsiveness in tendinopathy > 12 months likely stems from advanced degeneration (Lee et al., 2017), underscoring the need for phase-specific trials. However, these interpretations, remain preliminary given the limited evidence.

Hypothesis-generating subgroup analyses further explored ESWT types and Achilles tendinopathy subtypes but found no statistically significant differences. The effectiveness of focused *versus* radial ESWT remains debated: fESWT targets deeper tissues, potentially benefiting tendinopathy, while rESWT distributes energy more superficially (Auersperg & Trieb, 2020; van der Worp et al., 2013). Previous studies offer conflicting findings, with some favoring fESWT for deep-seated issues and others detecting no notable differences, highlighting the need for further research (Auersperg & Trieb, 2020; Şah et al., 2023). Non-insertional Achilles tendinopathy, prevalent among younger, active individuals, presented analytical challenges due to limited age-specific data. Theoretically, younger patients with healthier tendons and greater regenerative capacity may benefit more from ESWT, but current evidence is insufficient to confirm this (Pearce & Tan, 2016). Therefore, future studies should investigate these subgroups further.

In the sham-controlled design of this meta-analysis, placebo effects driven by patient expectations and contextual cues constitute an inevitable component. A systematic review of conservative treatments for plantar fasciitis explicitly quantified substantial placebo responses in sham ESWT cohorts (Viglione et al., 2023), emphasizing the need for rigorous evaluation of placebo effects. As outlined in Table 2, all eight RCTs employed targeted sham protocols designed to replicate authentic ESWT through measures such as zero-energy settings, disconnected probes, air-filled cavities, and interposed materials that dissipated waves while preserving auditory, positional, and tactile cues. Patient blinding was maintained across all trials, assessor blinding was achieved in seven trials, and formal validation of blinding success was reported in three trials, including less than 50% correct guessing in Pinitkwamdee et al. (2020), 85% of patients and 70% of providers providing incorrect guesses in Mansur et al. (2021), and 81% of patients providing incorrect guesses in Alsulaimani et al. (2025). Consequently, the overall risk of performance or detection bias arising from placebo effects in this synthesis remains reduced, supported by quantitative blinding validation, though minor uncertainties persist due to incomplete blinding validation in some studies.

Seven of the eight included RCTs incorporated co-interventions, primarily exercise protocols such as eccentric loading, stretching, and isometric training, applied equally to ESWT and sham arms. This balanced approach minimized confounding while aligning with evidence-based rehabilitation management of chronic Achilles tendinopathy. Evidence from the literature underscores that eccentric loading exercises, a cornerstone of rehabilitation, are supported by the highest level of evidence for improving tendon strength and reducing pain, particularly in early-stage tendinopathy (Silbernagel, Hanlon & Sprague, 2020). Stretching and isometric training further enhance tissue remodeling and pain management, contributing to a progressive loading program tailored to individual patient needs, as recommended for both midportion and insertional tendinopathy (Chen et al., 2022; Silbernagel, Hanlon & Sprague, 2020). A narrative review (Burton, 2022) of combined ESWT and exercise interventions for tendinopathy suggests potential synergy, with ESWT promoting tendon healing and exercise facilitating load-adaptive remodeling. However, it reports no consistent superiority of the combination over exercise alone across most tendinopathies. Clinical guidelines, including progressive loading protocols, advocate early rehabilitation with eccentric exercises, particularly for patients with symptom durations < 12 months, which aligns with our subgroup findings of transient pain relief (Silbernagel, Hanlon & Sprague, 2020). Therefore, the widespread use of co-interventions may mask ESWT’s isolated effects, contributing to the overall modest findings. Adding ESWT to standard rehabilitation involves notable costs ($300–$2,700 per course, depending on sessions and device), but the absence of economic data in the included studies precluded formal cost-effectiveness analysis. Future studies could incorporate such evaluations, including incremental cost-effectiveness ratios, alongside standardized protocols to refine ESWT-exercise integration for specific patient subgroups.

Given that statistically significant outcomes stem from hypothesis-generating subgroup analyses, our findings provide potential clinical insights rather than definitive medical recommendations. Patient selection seems critical, with individuals having symptom duration <12 months showing a higher likelihood of benefiting from ESWT. Early intervention may leverage the tendon’s residual healing mechanisms, potentially delaying progression to a chronic, degenerative state, though further investigation is warranted. The observed outcome variability highlights the need for standardized protocols, and the use of ESWT for non-insertional tendinopathy in younger, active individuals represents a promising underexplored area. These insights suggest that future studies could prioritize early intervention and tailored patient selection, such as for individuals with symptom duration <12 months. However, robust evidence from larger studies is needed to guide clinical decision-making.

This meta-analysis has several limitations. First, the small number of included studies and participants, coupled with limited representation of younger patients, restricts the strength of our conclusions. Second, although intergroup matching was maintained within individual studies, inter-study variations in patient demographics, ESWT parameters, and equipment may introduce heterogeneity that obscures the true effect of ESWT. Third, while co-interventions were standardized within each study’s groups, inconsistencies in their implementation across studies persist. Moreover, a prior review (Burton, 2022) indicated that combining exercises with ESWT is not consistently superior to exercises alone, which may confound the isolated impact of ESWT. Additionally, inconsistent reporting of activity levels, return-to-sport outcomes, and Achilles imaging across the studies precluded deeper analyses. Cost-effectiveness could not be evaluated due to the absence of economic data in the included trials. Furthermore, due to the limited number of studies and sample sizes, subgroup analyses comparing fESWT *versus* rESWT and non-insertional *versus* insertional chronic Achilles tendinopathy were highly constrained, preventing conclusions about outcome differences by modality or tendinopathy type. Finally, publication bias could not be formally assessed, as funnel plots and Egger’s test require at least ten studies per outcome for reliable interpretation.

## Conclusions

Primary findings, supported by low- to moderate- evidence, indicate that in patients with chronic Achilles tendinopathy, ESWT and sham ESWT demonstrated comparable safety profiles and no significant differences in pain reduction or functional improvement across follow-up periods. Hypothesis-generating subgroup analyses revealed a transient pain-relieving benefit of ESWT in patients with symptom duration < 12 months, while no differences emerged by ESWT type or tendinopathy classification. Overall, these findings, limited by small sample sizes and protocol heterogeneity, should be interpreted cautiously.

##  Supplemental Information

10.7717/peerj.20506/supp-1 Supplemental Information 1 Raw data

10.7717/peerj.20506/supp-2 Supplemental Information 2 PRISMA checklist

10.7717/peerj.20506/supp-3 Supplemental Information 3 Meta-analysis rationale

## Additional Information and Declarations

### Data Availability

The following information was supplied regarding data availability: This is a meta-analysis.
