# Supplementary material for: Extracorporeal shockwave therapy versus sham extracorporeal shockwave therapy for chronic Achilles tendinopathy: a meta-analysis of randomized controlled trials
Source: PeerJ. 2026 Jan 6;14:e20506. doi: 10.7717/peerj.20506 (PMC12786123; doi:10.7717/peerj.20506)
Supplement: Supplemental Information 3 [file peerj-14-20506-s003.docx]

This meta-analysis is for clinical rehabilitation therapists (physiotherapists) and orthopedic physicians. They need evidence to guide treatment for chronic Achilles tendinopathy, especially concerning Extracorporeal Shockwave Therapy (ESWT) effectiveness versus sham treatments. By focusing on randomized controlled trials with sham controls, this study provides high-quality evidence to help these clinicians make informed decisions on ESWT use, patient selection based on symptom duration, and patient counseling.
